# Fault diagnosis for wind turbines with graph neural network model based on one-shot learning

**DOI:** 10.1098/rsos.230706

**Published:** 2023-07-05

**Authors:** Shuai Yang, Yifei Zhou, Xu Chen, Chuan Li, Heng Song

**Affiliations:** ^1^ Chongqing Technology and Business University, National Research Base of Intelligent Manufacturing Service, Chongqing, People’s Republic of China; ^2^ School of Management Science and Engineering, Chongqing Technology and Business University, Chongqing, People’s Republic of China; ^3^ Institute of Management Research, China Railway No. 4 Engineering Group, Shanghai 201600, People’s Republic of China

**Keywords:** wind turbine, fault diagnosis, deep learning, graph neural network, convolutional neural network, one-shot learning

## Abstract

Because of the harsh working environment, there is usually a lack of effective data from the gearboxes of wind turbines for fault classification. In this paper, a fault-diagnosis model based on graph neural networks and one-shot learning is proposed to solve the problem of fault classification with limited data. In the proposed method, the short-time Fourier transform is used to convert one-dimensional vibration signals into two-dimensional data, then feature vectors are extracted from the two-dimensional data, and small-sample learning is achieved. An experimental rig was built to simulate the real working scenario of a wind turbine, and the results indicate the high classification accuracy of the proposed method. Furthermore, its effectiveness is verified in comparisons with Siamese, matching and prototypical networks, with the proposed method outperforming all of them.

## 1. Introduction

Wind power has played a leading role in renewable energy for the past four decades and will potentially be a major form of energy in the near future. Based on its growth, wind energy has also been one of the fastest-growing forms of energy because of its reliability and cost-effectiveness [1]. However, the extreme operating conditions of wind turbines expose them to high-frequency failure, and so it is critical to minimize property losses and prevent casualties through early detection of these impending faults [2].

As the most important transmission part of a wind turbine, the gearbox has been shown to account for the highest failure rate [3]. In recent years, gearbox diagnosis has been the subject of much research, leading to widespread interest in data-driven gearbox fault diagnosis. The general steps of this type of approach are: (i) signal collection, (ii) data processing and feature extraction, and (iii) fault detection [4]. Among various data-driven methods, deep learning models such as deep belief networks (DBNs) [5] and convolutional neural networks (CNNs) have been applied widely to the diagnosis of mechanical faults.

Regarding the DBN approach, Wang *et al.* [6] proposed an algorithm based on local tangent space alignment and a DBN, and experimental results indicated that the proposed method not only reduces the dimension of high-dimensional data but also improves the classification accuracy of the intelligent diagnosis model. Also, Dai *et al.* [7] proposed a new transformer fault-diagnosis method based on a DBN with rectified linear units (ReLU-DBN), which improved the accuracy of fault diagnosis through multi-dimensional and multi-layer mapping. However, the weak anti-noise ability and poor stability of DBNs mean that their industrial applications are limited.

First proposed by LeCun *et al.* [8] to apply backpropagation to handwritten zip codes, CNNs as typical representatives of deep learning have made significant progress in image classification, object detection and other fields [9]. Lee *et al.* [10] proposed an induction-motor fault-diagnosis system based on a CNN model, and experimental results confirmed that the proposed method is suitable for diagnosing rotor and bearing faults in induction motors. Zhang *et al.* [11] improved the performance of typical CNNs by optimizing the impact of the ordering of input measurement parameters, and in simulation experiments the proposed method performed well in terms of precision, stability and comprehensibility. CNNs have achieved good results in many fields, such as image recognition and natural language processing, but their efficient processing is limited to regular Euclidean data such as grids and sequences.

However, in non-Euclidean data, the number and order of neighbouring nodes of central nodes are not fixed, so it is difficult to define convolution kernels for such data. The most typical form of non-Euclidean data is the graph structure, and hence graph neural networks (GNNs) came into being. The data objects processed by GNNs are graph data with irregular structures, so in practice various non-Euclidean distance problems can be abstracted into graph structures. Hua *et al.* [12] proposed a fault-prediction method for a distributed network based on a GNN, which improved the accuracy of the algorithm by 3%. Shanshan *et al.* [13] proposed a fault-diagnosis method for rolling bearings based on a CNN and GNN, which involves converting one-dimensional feature data into graph data and then using a three-layer GNN; results showed that the method has a fault-diagnosis accuracy of more than 90%.

However, all the aforementioned deep-learning models require a large amount of data for training to reach a high accuracy [14]. In 2003, Li *et al.* [15] proposed one-shot learning (OSL); most machine-learning-based object-classification algorithms require hundreds or thousands of images or very large databases for training, whereas the main goal of OSL is to learn object classification from one or very few training images. When aimed at classification problems, OSL can achieve high classification accuracy with a small amount of training data, such as one or a few visual images [16]. Because of the harsh working environment, effective data from the gearboxes of wind turbines are usually limited, so this paper proposes a GNN model based on OSL for fault classification.

The remainder of this paper is structured as follows. The methodology of the proposed method is introduced in §2, and it is evaluated experimentally in §3. Results are presented and discussed in §4, and conclusions are drawn in §5.

## 2. Methodology

### 2.1. Convolutional neural network extraction of vector features

**Figure 1.**
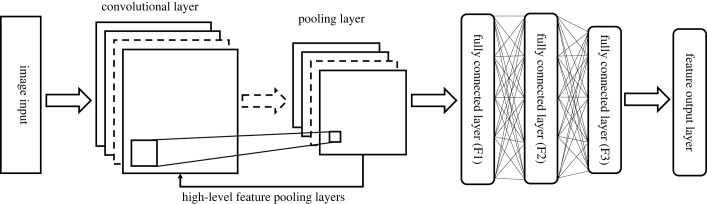
Convolutional neural network (CNN) feature-recognition framework.

Image feature recognition and retrieval are realized by extracting vector features with a CNN, and the process is shown in figure 1. A CNN was chosen for feature extraction and classification because of its shared convolutional kernel and high processing power, and the steps are as follows. (i) *Input*. The input of a CNN is an image, which is usually a multi-channel matrix. (ii) *Convolutional layer*. This consists of a series of convolutional kernels, each of which slides over the input image and performs convolutional operations on local regions. (iii) *Pooling layer*. This is used to reduce both the spatial dimensions of the feature map and the computational effort by extracting the main features by pooling the maximum or average values of the local areas. (iv) *Fully connected layer*. After multiple convolutions and poolings, the feature map is converted to vector form and connected to the fully connected layer, which is usually a multi-layer perceptron (MLP) that maps feature vectors to output classes or makes predictions for other tasks. (v) *Output layer*. The final layer is the output layer, which selects the appropriate activation and loss functions according to the specific task and produces the corresponding outputs [17].

**Figure 2.**
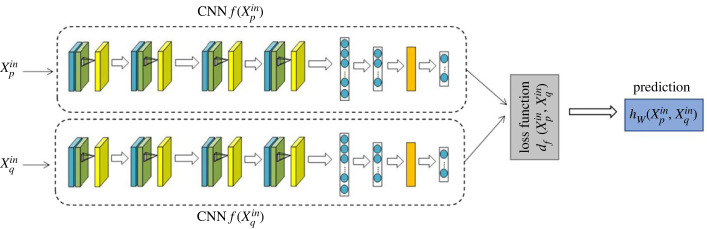
CNN feature-extraction prediction map.

During the feature-extraction process of the CNN, the convolution window slides over the image, and the elements in the window are convolved. All the outputs generated during the traversal process form feature maps by position, and how many feature maps each layer must design depends on how many features must be learned. The quality of the design of the number of feature maps directly affects the final network performance, so we use a CNN approach for feature extraction, as shown in figure 2, which consists of an input layer, two CNNs, a distance metric and an output layer. In the output layer, we represent $x_{p}^{in}$, $x_{q}^{in}$ as a pair of samples of the same or different classes, where $x_{p}^{in}$, $x_{q}^{in}$ are the two data as input.

The two identical CNNs are connected by a distance metric between their outputs, and they use the same distance parameters and weights. Convolutional layers with 3–3 filters use ReLU activation functions [18]. Additionally, batch normalization is used to speed up learning by normalizing the input layer, and dropout is added for normalization. Flatten is used to convert two-dimensional matrices to vectors, and the distance metric is calculated as a vector as

2.1 $$d_{f}\;(x_{p}^{in},x_{q}^{in})=|f\;(x_{p}^{in})-f\;(x_{q}^{in})|,$$

where *f*( · ) is the CNN and |*x*| is the element-wise absolute value of the vector. The output of the proposed OSL model represents the similarity probability between two input samples and is obtained by

2.2 $$h_{w}(x_{p}^{in},x_{q}^{in})=\sigma \;(\mathrm{FC}(d_{f}\;(x_{p}^{in},x_{q}^{in}))),$$

where *σ*( · ) is a sigmoid function, FC( · ) is a fully connected layer and *w* is a vector of learnable weights used to collect each parameter that should be determined in the model.

### 2.2. Data preprocessing with short-time Fourier transform

The Fourier transform (FT) was once the most widely used method for signal processing. However, the fast FT can only reflect the character of the signal in the frequency domain, so instead the short-time FT (STFT) is chosen to treat the non-stationary signal as a series of short-time signals and divide the signal into several small intervals. A sliding time window is used to calculate the frequency spectrum to determine the frequency within a certain time interval, based on the traditional FT [19].

The STFT of a continuous-time signal *S*(*t*) is expressed mathematically as

**Figure 3.**
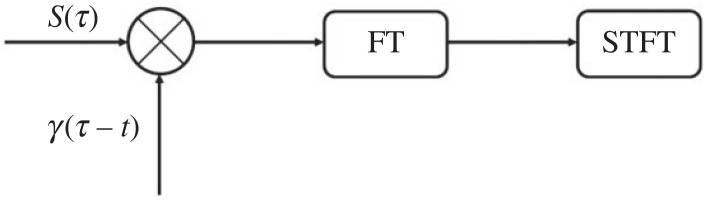
Schematic of implementation of continuous-time short-time Fourier transform (STFT).

2.3 $$\begin{array}{rl} \mathrm{STFT}(t,\omega ) & =\int_{-\infty }^{+\infty }S(\tau ),\gamma (\tau -t)\;e^{-j\omega \tau }\;d\tau \\ & =\langle S(\tau ),\gamma (\tau -t)e^{-j\omega \tau }\rangle \\ & =\langle S(\tau ),\gamma_{t,\omega }(\tau -t)\rangle , \end{array}$$

where *γ*(*t*) is a window function that is usually real and even. In addition, for the continuous-time signal *S*(*t*), *ω* is the angular frequency and *j* is the imaginary unit, with *j*^2^ = −1. Finally, $c_{n}=\langle S,\psi_{n}\rangle =\;\int_{-\infty }^{+\infty }S(t)\Psi_{n}^{*}(t)\;dt$ is called the inner product or dot product. The STFT of the continuous-time signal *S*(*t*) is realized as shown schematically in figure 3.

In the time domain, the window function is used to cut off the signal *S*(*t*). The window is moved to the starting point of the signal, at which time the centre position of the window function is at *t* = *t*_0_, and the signal is windowed as

2.4 $$y(t)=S(t)\times W(t-t_{0}),$$

where *t* is the time translation parameter, *S*(*t*) is the original signal and *W*(*t* − *t*_0_) is the sequence of real window functions. The intercepted signal can be obtained by multiplying the window function and the original signal *y*(*t*), with the intercepted signal *y*(*t*) being the signal of execution time corresponding to *t* [20].

### 2.3. Fault classification with graph neural network based on one-shot learning

A GNN is a graph domain information processing method based on deep learning. Because of their good performance and interpretability, GNNs have become widely used for graph analysis. Many important datasets exist in the form of graphs or networks, and if neural networks and graph analysis can be combined, then many problems will be solved. The most important components of a graph structure are the vertices and edges, which are represented as

2.5 G=(V,E),

where *G* represents the graph structure, *V* represents the nodes and *E* represents the edges connecting the nodes.

**Figure 4.**
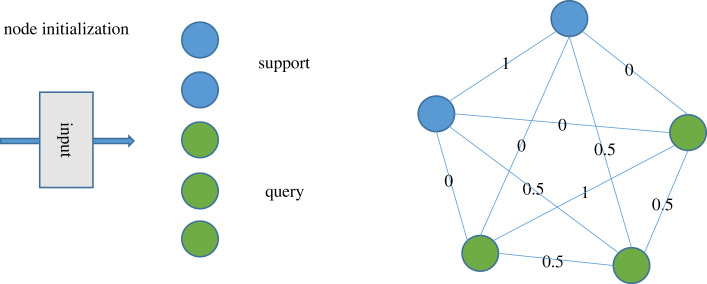
Schematic of GNN edge node initialization.

In the classification task, the small-sample data are divided into a support set and a query set, and as shown in figure 4, the GNN constructs a graph structure for classification. It establishes connections between all the samples, with the edge labels assigned explicitly in the initialization stage (i.e. edge weight initialization) and then updated explicitly. Finally, the edge weights are used as the basis for classification to discriminate among the unknown samples, which is also a GNN. In terms of how this network differs from other networks, the general network updates the nodes, i.e. the features of the image for classification, and an edge has only one of two values, i.e. 0 or 1. The adjacency matrix is shared in each iteration of the model and is not updated, but the GNN finally uses the edge weights as the main classification basis, and only the initial value of the edge weight of 0.5 can be changed and updated [21].

Herein, each node represents an image, and the weight of each edge represents the relationship (distance or similarity) between two images. The weights are calculated specifically as

2.6 $${\tilde{A}}_{i,j}^{\left(k\right)}=\varphi_{\tilde{\theta }}(x_{i}^{\left(k\right)},x_{\;j}^{\left(k\right)})={\mathrm{MLP}}_{\tilde{\theta }}(abs(x_{i}^{\left(k\right)}-x_{\;j}^{\left(k\right)})),$$

where *x*^*k*^ is received as the input of a GNN layer, and φ = MLP is a symmetric function parametrized with (for example) a neural network. Herein, a stacked MLP is considered after the absolute difference between two vector nodes.

**Figure 5.**
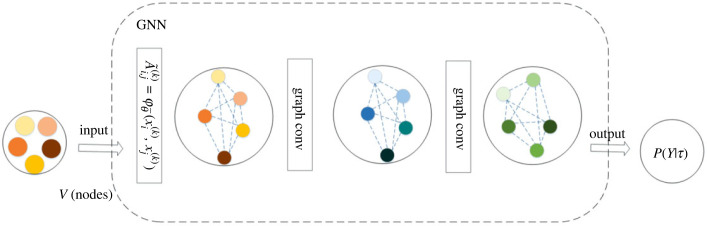
Graph neural network (GNN) model structure.

The adjacency matrix is computed before every convolutional layer. The input is preprocessed data, and the fault data may include graph data, with each node representing graph data, followed by the corresponding weight calculation of the input data, and then the output of the model is sent through a multi-layer GNN to determine the fault category, which is shown in figure 5.

**Figure 6.**
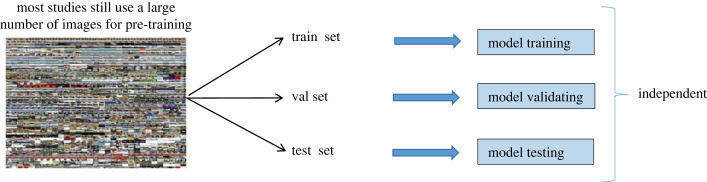
Diagram of general deep network.

As shown in figure 6, when the training starts, the dataset is divided into three parts, with each part input separately into the model; this differs from the general deep network that uses training and testing separately. During small-sample classification, most methods use all the samples at once to construct a graph structure, regardless of whether the labels are known or unknown, so from the perspective of sample utilization, this construction method can be used to a certain extent, but it often takes a long time to train [22].

**Figure 7.**
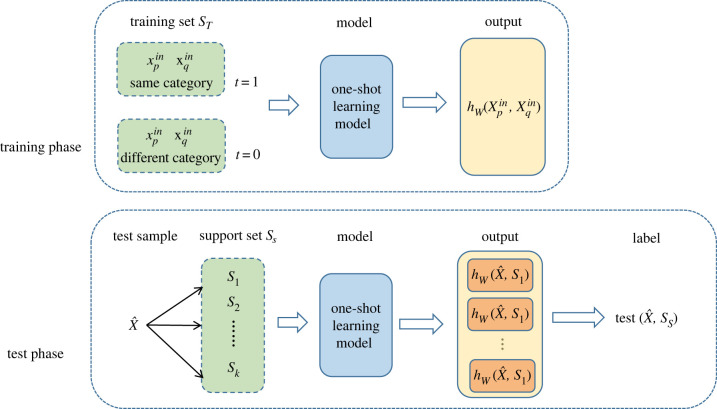
One-shot learning strategy.

So, as mentioned before, OSL is used to provide only one or a few training samples; i.e. it needs only a small number of labelled samples for learning. Figure 7 shows the architecture of the proposed OSL model. In the training phase, the inputs are pairs of samples of either the same class or different classes, and the output is the probability of similarity between the inputs [23].

## 3. Experimental verification

**Figure 8.**
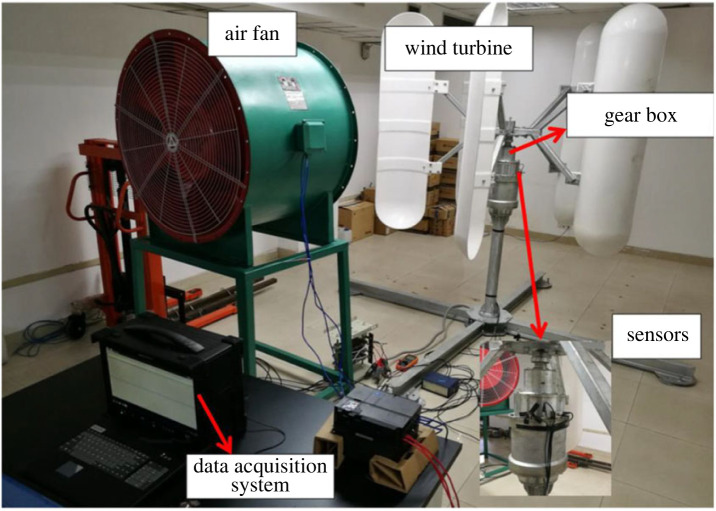
Experimental set-up.

To verify the effectiveness of the proposed method, experiments were carried out at Chongqing Technology and Business University. Figure 8 shows the experimental apparatus, which comprised a wind turbine, a fan, a data acquisition system, sensors and a workstation. The fan was used to simulate the wind in the natural environment, and the generated wind rotated the turbine blades to drive the rotation of the gears. The vibration signals of the vibration *X* and *Y* axes were each collected by an acceleration sensor and transmitted to the workstation through the high-speed data acquisition system.

The vibration data were obtained from the sun gear in the gearbox, which had eight different conditions, i.e. (1) healthy, (2) light pitting, (3) medium pitting, (4) missing half tooth, (5) missing full tooth, (6) medium double pitting, (7) pitting + missing tooth, and (8) 0.3–0.5 worn. The details of the sun gear are shown in table 1. Each test involved a sampling time of 20 s and was repeated 10 times. Three different frequencies (35 Hz, 40 Hz and 50 Hz) were selected for each fault, and all the loads were zero. The vibration signals for the *X* and *Y* axes were collected by accelerometres with a sampling frequency of 100 kHz and combined different faults.

**Table 1.** Condition patterns of sun gear used in experiment.

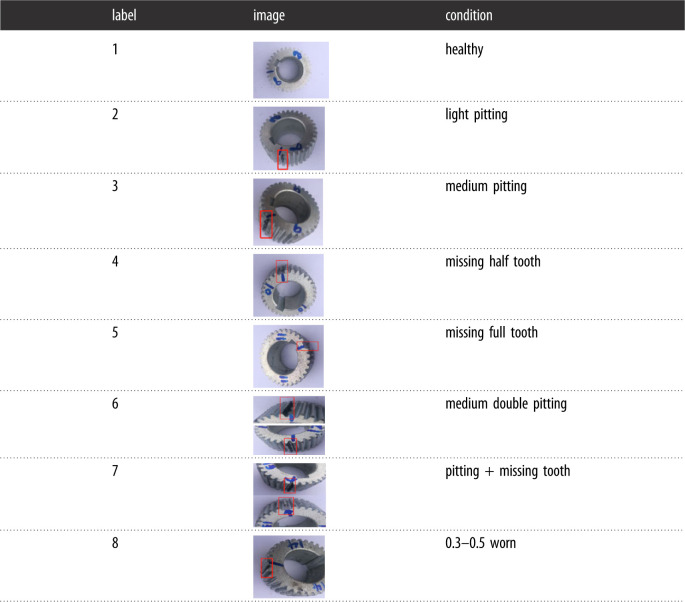

The loss and accuracy functions of the model are

3.1 $$\mathrm{cross}\_\mathrm{loss}=-\sum_{k}t_{k}\log y_{k}$$

and

3.2 $$\mathrm{accuracy}=\frac{\mathrm{TP}+\mathrm{TN}}{\mathrm{TP}+\mathrm{TN}+\mathrm{FP}+\mathrm{FN}},$$

where *y*_*k*_ is the output of the neural network and *t*_*k*_ is the correct solution label. True positive (TP) means correct division into positive samples, true negative (TN) means correct division into negative samples, false positive (FP) means incorrect division into positive samples and false negative (FN) means incorrect division into negative samples.

**Figure 9.**
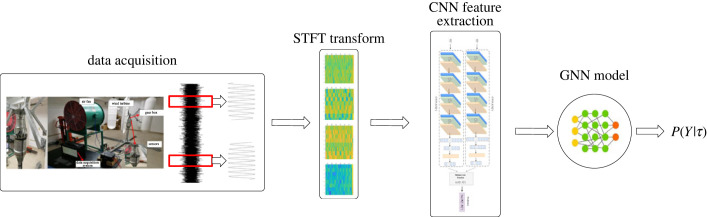
Flowchart of proposed method.

All raw data are converted into image data, as shown in figure 9. The output vibration signal is first subjected to STFT to convert the one-dimensional signal into two-dimensional data. The output data are then input into the CNN for feature extraction, and finally each image is processed into a 64-dimensional feature vector. Finally, this is input to the neural network, using leaky ReLU as the activation function and using the cross entropy loss function as the loss metric, and its accuracy is calculated.

## 4. Main results

**Figure 10.**
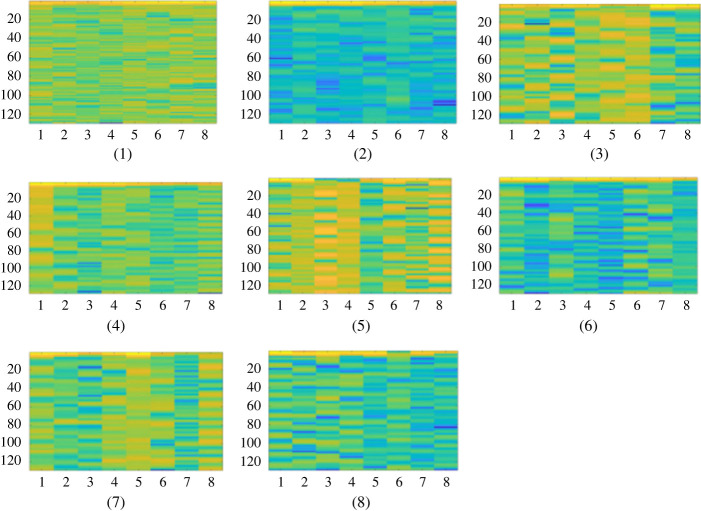
Transformed graphs under different conditions: (1) healthy; (2) light pitting; (3) medium pitting; (4) missing half tooth; (5) missing full tooth; (6) medium double pitting; (7) pitting + missing tooth; (8) 0.3–0.5 worn.

In the experiments, 9600 samples were collected and preprocessed using STFT in Matlab to convert one-dimensional signals into two-dimensional images. To illustrate the signal of the fan in different states, figure 10 shows the collected vibration signals in eight states converted into two-dimensional data.

For comparison, methods other than the proposed OSL image classification method were used to classify faults under the same dataset, with the proposed method found to be superior to a Siamese network, a matching network, and a prototypical network. These were chosen for comparison for the following reasons: Siamese networks are simple and effective and are used widely in computer science, face recognition, finance and other fields; a matching network combines an attention structure and a memory network to build a fast learning network; a prototypical network is an algorithm for semi-supervised learning and is an effective few-shot learning model with fewer parameters than traditional network models.

**Figure 11.**
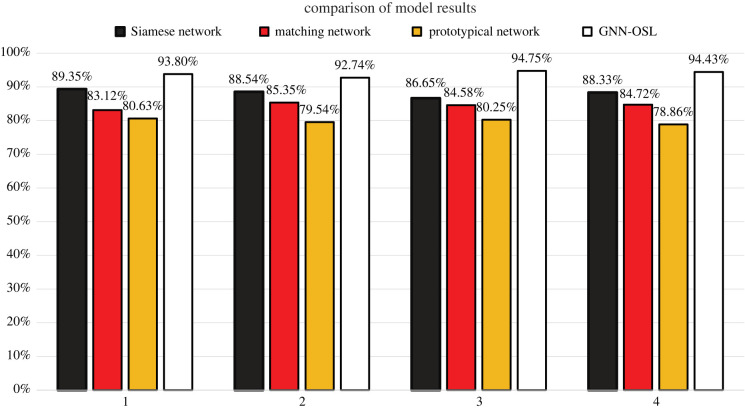
Comparison of model results.

Table 2 and figure 11 compare the accuracies of the different models, whose parameter values are given in table 3. All 9600 collected samples were given to the proposed model, and its highest accuracy was 94.7475% in the results of five runs. Meanwhile, the accuracies of the Siamese, matching and prototypical networks were only 89.35%, 85.36% and 80.63%, respectively, so the proposed method is the most accurate.

**Table 2.** Comparison of results under different models.

model	accuracy
Siamese network	89.3501%
matching network	85.3600%
prototypical network	80.6250%
GNN-OSL	94.7475%

**Table 3.** Parameter values of each model.

model	learning rate	batch_size	iterations
Siamese network	0.007	32	10 000
matching network	0.007	5	10 000
prototypical network	0.0001	64	10 000
GNN-OSL	0.007	32	10 000

## 5. Conclusion

Herein, a fault-diagnosis GNN model based on OSL was proposed for classifying the faults of wind-turbine gearboxes. In most cases, there is a lack of effective data from gearboxes for fault classification, but the proposed method can obtain a high-accuracy training model with small datasets. This method converts one-dimensional vibration signals into two-dimensional data by using STFT. After extracting feature vectors from the two-dimensional data, the method implements OSL. To verify the proposed method, an experimental rig was built to simulate the real working scenario of a wind turbine. With 10 000 iterations and a learning rate of 0.007, the classification accuracy of this proposed method can reach 94.75%. Finally, to further verify the effectiveness of the proposed method, it was compared with Siamese, matching and prototypical networks under the same number of iterations. The accuracies of those networks were only 89.35%, 85.36% and 80.63%, respectively, so the proposed method is the most accurate, which indicates its effectiveness.

Despite the effectiveness of the proposed method, an issue remains to be addressed in future work. The experiment reported herein verified the applicability of the model only for wind turbines, and its effectiveness for other rotating equipment remains to be seen. In future work, we will incorporate transfer learning into the proposed method and apply it to various rotating equipment under different operating conditions in order to verify further its universality.

## Data Availability

This article has no additional data.
